# 

**Published:** 1887-07-09

**Authors:** 


The Hospital.

An Institution, Family, and Parochial Journal of Hospitals, Asylums, and all Agencies for the Care of the Sick, Criticism and News.

SATURDAY, JULY 9TH, 1887.

### NOTICE TO CORRESPONDENTS.

All correspondence must be written on one side of the paper only.
We cannot undertake to return MSS. not used.
**Communications,** whether intended for insertion or for private information, must be authenticated by the names and addresses of the writers, not necessarily for publication.
**Local papers** containing reports or news paragraphs **should be marked.**
It is especially requested that early intelligence of **local events of interest,** or which it may be thought desirable to bring under notice, may be sent direct to the Editor.
Communications respecting editorial matters should be addressed to the Editor, Norfolk House, Norfolk Street, Strand, London, W.C.

### NOTICE TO ADVERTISERS.

*To insure prompt insertion, all Advertisements for* THE HOSPITAL *must be sent in direct to the Advertising Agent, Mr. A. P. Watt,* 2, *Paternoster Square, E.C.*

### THE QUILTER PRIZE.

WE regret to say that very few papers have been sent in for this competition; that in most cases the conditions have not been fulfilled; and that the adjudicators do not consider any one paper of sufficient merit to warrant the giving of the first prize. In deference to wishes expressed they propose to reconsider their decision, with a view to the awarding of second or third prizes, if any paper should be found to reach a fair standard of merit.

### THE LITERARY PRIZE.

A PRIZE of **Two Guineas** will be given every month for the best story or incident based upon hospital, or asylum, or student life, or any incident connected with the professions of medicine or nursing.

### THE CHILDREN'S CORNER.

**Fourth Quarterly Prize Competition.**
Began 2nd July, 1887 ; ends 24th Sept., 1887.

PRIZES.
FOR MOST CORRECT ANSWERS TO PUZZLES.

| | | | |
|---|---|---|---|
| 1st .. .. | Thirty Shillings | 3rd .. .. | Ten Shillings |
| 2nd .. .. | Twenty „ | 4th .. .. | Five „ |

FOR NEATEST ANSWERS TO PUZZI ES.
Five Shillings.
FOR BEST LITTLE LETTERS.
Ten Shillings.

**Puzzles—Second Week.**

No. 5. BIBLICAL CHARADE. My first is a [illegible] in Switzerland, my second a preposition, and my whole i[illegible]he name of a Jewish High Priest.

No. 6. DECAPITATION.

I am of every size and shape,
And of every colour too;
I am down in the sea, and up in a tree,
And am sometimes found in your shoe.
Cut off my head and you see me no more,
Yet still I am to be heard
In the sounding bell, in the wild sea's roar,
And the voice of the singing bird.
Remove now my tail, 'tis easily done,
When, wonderful to relate,
To your surprise, though smaller in size,
I am greatly increased in weight.

No. 7. SQUARE WORD. 1. A title. 2. One. 3. A large stove. 4. A volcanic mountain.

No. 8. FLORAL CAARADE.

My second a part of my first will be found ;
In the green grassy mead my whole doth abound.

**Letters—Second Week.**

Next week tell me something about your favourite *Wild-flowers*, and where they are to be found.

**Results of Thirteenth Week—Puzzles.**

No. 51. HISTORICAL ACROSTIC.

| | | |
|---|---|---|
| S | carboroug | H |
| T | yn | E |
| O | rga | N |
| N | urember | G |
| E | lb | E |

No. 52. RIDDLE. Your name.

No. 53. TOWNS IN IRELAND ENIGMATICALLY EXPRESSED. 1. Bel-fast. 2. Cork. 3. Water-ford. 4. Band-on.

No. 54. CHARADE. Bull-dog. I have received several good solutions of this Chara le.

*All right*—Skye, Scrooge, Alsie, Joe, Dot, Gom ; *Three right*—Mikado, Bohemian Girl, Robert Epton, A. G. S. McCulloch, Tim, Umslopogaas, A. C. K., Nordisa, Princess Ida : *Two right*—The Eda, Edith.

**Letters.**

Some of these will be found in another column. Bohemian Girl, Mikado, and Skye also send me interesting letters.

One mark each :— Princess Ida, Edith, Eric, Ellie, Skye Bohemian Girl, Mikado.

**Notices to Correspondents.**

NORDISA and SKYE.—Your solution was a good one, but mine is better. Other correspondents gave the same, which was not accepted.

**Results of Third Quarterly Competition.**

The contest this quarter has been an exceedingly close one, and *three* of my little friends have succeeded in gaining the highest score, namely—

DOT, Score 49 (Miss Norah Kellner, 1, Stanwick Street, Tynemouth, Newcastle-on-Tyne, aged 15).

NORDISA, Score 49 (Miss Jessie Glanville, 12, Sydenham Terrace, Newcastle-on-Tyne, aged 13).

SKYE, Score 49 (Miss Ella R. Jefferiss, 4, Milton Terrace, New Road, Chatham, aged 14).

I have therefore divided the First Three Prizes equally between them.

The Fourth Prize is also a tie, and will be divided between—

A. C. K., Score 48 (Master Arthur Clifton Kelly Ellesmere, Gratwicke Road, Worthing, aged 10).

SCROOGE, Score 48 (Miss Constance L. Mills, 7, Charles Street, Pendleton, aged 13).

It is matter for congratulation to see how keenly these prizes are contested. The industry, perseverance, and knowledge shown by the majority of our young friends is very gratifying, and the improvement which the answers of several small people exhibit, is not the least satisfactory feature of these Competitions. We hope many more children will enter for the prizes this quarter.

LETTERS.

MIKADO, Score 13 (Miss F. H. Gibson, 12, Upper Tichborne Street, Leicester) gains the prize of 10*s*.

NEATNESS.

The prize, 5*s*., for general neatness in the papers sent in, has been gained by
BOHEMIAN GIRL (Miss C. M. Gibson, 12, Upper Tichborne Street, Leicester, aged 13).

Answers, having the actual name and address (to which a pseudonym may be added for publication), must have "The Children's" Coupon attached (see advertisement pages), and be addressed to the "PUZZLE EDITOR," THE HOSPITAL, Norfolk House, Norfolk Street, Strand, W.C., not later than Thursday next.

### AMUSEMENTS WITH PROFIT.

### PRIZE COMPETITIONS.

**Quarterly Prize Competition.**
Began 23rd April ; ends 16th July.

**A Prize of Three Guineas**
will be awarded to the Competitor who shall have sent in the largest number of correct or best answers. No one will be permitted to win the Three Guinea Prize twice in any one year, but we shall award annually an additional
**Prize of Five Guineas**
to the competitor who shall have sent in the largest number of correct or best answers during the twelve months.

**No. 22. Double Acrostic.**

The initials give the name of an eminent politician, and the finals that of a distinguished statesman and politician.

1. A recent exhibition held in London.
2. The goddess of health.
3. The reverse of useless.
4. An eminent modern poet and painter.
5. Confusion.
6. A sacred mount of the Old Testament.
7. A word denoting " done in haste."
8. A son of toil.
9. A cutter of precious stones.

**No. 23 Enigma.**

Old Doctor Brown
In his dressing gown,
Was ensconced close in front of the fire,
With my *first* on his head,
Well armed, it is said,
By the Doctor's especial desire—
Whate'er his sensation,
He feared not starvation,
As he sat in the warmth of the blaze,
While eating my *second*,
That's usually reckoned
Nutritious in medical phrase.
But between you and I,
As stories will fly,
His illness was under control,
And although he pretended
His days were near ended,
He only laid up for my *whole*.

**Answers.**

No. 19. DOUBLE ACROSTIC.

| | | |
|---|---|---|
| T | angen | T |
| H | ematit | E |
| E | pigra | M |
| R | ap | P |
| M | aclur | E |
| O | leande | R |
| M | alt | A |
| E | rfur | T |
| T | ol | U |
| E | cuado | R |
| R | obespierr | E |

Correct answers have been received from Aralc, Dawnlover, Canice, Gretta, Letheàdor, K. M., Mater, A. M. E., May.

NO. 20. MATHEMATICAL QUESTION.

The answer to this is 18, or any multiple of 18.

Correct answers have been received from Gretta, Letheàdor, Ellice, K. M., Canice, Aralc, Dawnlover, Mater, Tabs, May A. M. E.

Answers must be addressed to THE PRIZE EDITOR, AMUSEMENTS WITH PROFIT, THE HOSPITAL, Norfolk House, Norfolk Street, Strand, W.C., not later than the *first* post on Friday next. The full name and address must in all cases be given, but a *nom de plume* may be added for publication. Only one side of the paper should be written on. The "Amusements with Profit" Coupon (see advertisement pages) must be enclosed with replies.

### The In- and Out-Patients' Hospital Diary.

This Diary is so arranged as to make some portion of it appear every week, and the whole in each monthly part. It has been very difficult to compile, and corrections will at all times be gratefully received. It has been suggested that similar information about the larger Provincial and Scotch Hospitals would be useful to many readers. We should be glad to hear if this is felt to be the case.

### I.—GENERAL HOSPITALS.

| Hospitals and Terms of Admission. | Physicians, Surgeons, and Medical Officers, with Days and Hours of Attendance. |
|---|---|
| Charing Cross Hospital, West Strand, W.C. Sec., Mr. A. E. Reade<br>Urgent cases are immediately admitted as in-patients. Subscriber's letter expected in other cases<br>Out-patients admitted without subscriber's letter first time; for continued attendance they must be obtained from the Secretary | *In-Physicians*, Drs. Pollock, M. Th.; Green, W. S.; Bruce, Tu. F. Hour, 1.30<br>*In-Surgeons*, Messrs. Barwell, M. Th.; Bellamy, Tu. F.; Bloxam, W. S. Hour, 2<br>*Out-Physicians*, Drs. Wilcocks, M.Th.; Abercrombie, Tu. F.; Lubbock, W. S.; Murray, W. S. Hour, 1.30<br>*Out-Surgeons*, Messrs. Cantlie, M.Th.; J. H. Morgan, Tu. F.; Stanley Boyd, W. S. Hour, 1.30 |
| "Dreadnought" Hospital (Seamen's Hospital Society), Greenwich, S.E. Sec., Mr. W. T. Evans<br>Entirely free to seamen and to all accident cases | *In- and Out-Physicians*, Drs. Curnow, Tu. Th. S.; H. T. Griffiths, M.W.F. Daily, between 9 and 3<br>*In- and Out-Surgeons*, Messrs. W. J. Smith, G. R. Turner. Daily, between 9 and 3 |
| French Hospital, 10, Leicester Place, Leicester Sq., W.C. Sec., Mr. E. Rimmel<br>Free to urgent cases and to all foreigners speaking French | *In-Physician*, Dr. Vintras, Tu. S. 10<br>*In-Surgeons*, Sir W. MacCormac, Tu. 9; Mr. J. Késer, W. F. 10<br>*Out-Physician*, Dr. Vintras, Tu. S. 10<br>*Out-Surgeons*, Messrs. H. de Méric, M. Th. 10; J. Késer, W. F. 10 |
| German Hospital, 113, Dalston Lane, E. Sec., Mr. C. Feldmann<br>Free to natives of Germany, and others speaking German, and to all accidents and emergencies. Others by Governors' letters<br>——— Eastern Branch, 49, Mansell Street, Aldgate, E.<br>——— Western Branch, 259, Oxford Street, W. | *In-Physicians*, Drs. Weber, M. T. 2; Port, Tu. F. 9 a.m.<br>*In-Surgeons*, Drs. Lichtenberg and Burger, W. S. 2<br>*Out-Physicians*, Drs. Tuchmann, Tu. W. 2; Ludwig, M. Th. 2; Keller, Tu. W. F. 2; Philippi, M. Th. F. 2 (*Men*, Tu. Th.; *Women*, M. W. F.)<br>*Out-Physician*, Dr. C. Harrer, Tu. F. 8 to 10 a.m.<br>*Out-Physician*, Dr. M. Castaneda, M. T. 8 to 10 a.m. |
| Great Northern Central Hospital, Caledonian Road, N. Sec., Mr. W. T. Grant<br>Free to all, without letters of recommendation | *In-Physicians*, Drs. Cholmeley, Cook, and Burnett, M. Tu. W. Th. F. 2<br>*In-Surgeons*, Messrs. W. Adams, W. Spencer Watson, and J. Macready, M. Tu. W. Th. F. 2<br>*Out-Physicians*, Drs. Beale, M. Th. 2; Beevor, Tu. 2, S. 10<br>*Out-Surgeons*, Messrs. Lockwood, M. Th. 2; Makins, Tu. F. 2 |
| Guy's Hospital, St. Thomas's Street, S.E. Supt., Dr. C. J. Steele<br>Patients are admitted, if beds are available, without letters of recommendation, on application at the Hospital. Out-patients are seen free without letters of recommendation | *In-Physicians*, Drs. Pavy, Tu. F. Pye-Smith, M. Th. S.; Taylor, M. Tu. Th. F. Hour, 1.30<br>*In-Surgeons*, Messrs. Bryant, M. Th. Durham, M. Th. F.; Howse, W. S.; D. Colley, M. Th. Hour, 1.30<br>*Out-Physicians*, Drs. Carrington, W.; Hale White, M.; Goodhart, M. Tu. Th. F. Hour, 12.30<br>*Out-Surgeons*, Messrs. Lucas, Th.; Golding Bird, S.; Jacobson, W.; Symonds, M. Hour, 12.30 |
| King's College Hospital, Portugal Street, W.C. Sec., Mr. T. Mosse Macdonald<br>Accidents and urgent cases free at all times. Other cases by Governors' letters, but where not obtainable, application may be made to the Secretary | *In-Physicians*, Drs. Beale, Duffin, Yeo, Tu. W. F. 1.30; Tu. W. F. S. 2<br>*In-Surgeons*, Mr. Wood, Sir J. Lister, Messrs. H. Smith, W. Rose. Daily, 1.30<br>*Out-Physicians*, Drs. Ferrier, J. Curnow, N. I. C. Tirard. Daily, 1.<br>*Out-Surgeons*, Messrs. W. Rose, W. Cheyne, A. B. Barrow. Daily, 1. |
| London Hospital, Whitechapel Road, E. Sec., Mr. A. Haggard<br>Accidents and urgent cases free; out-patients for dental and maternity departments need no recommendation; other cases by subscriber's order. Out-patients are required to attend only once, unless otherwise directed by the Physician or Surgeon | *In-Physicians*, Drs. Mackenzie, Tu. F.; Down, Tu. F.; H. Jackson, M. Th.; Sutton, M. Th.; Fenwick, Tu. F. 1.30<br>*In-Surgeons*, Messrs. Rivington, Tu. F.; Couper, Waren Tay, M. Th.; McCarthy, W.S.; Treves, Tu.F. Hour, 1.30<br>*Out-Physicians*, Drs. Sansom, Th.; M. Ralfe, M. Th.; Turner, Tu. F.; Warner, Tu. F.; Gilbart Smith, W. S. J. Anderson, W. S. Hour, 1.30<br>*Out-Surgeons*, Messrs. Mansell-Moullin, M. Th.; Eye, M. W.; Reeves, Tu. S.; H. Fenwick, Tu.F. Hour, 1.30 |

| Hospitals and Terms of Admission. | Physicians, Surgeons, and Medical Officers, with Days and Hours of Attendance. |
|---|---|
| London Temperance Hospital, Hampstead Road, N.W.<br>For treatment without alcohol. By letter or small payment | *In- and Out-Physicians*, Drs. J. Edmunds, M.; J. J. Ridge, Tu. F. Hour, 1.30<br>*In- and Out-Surgeons* Mr. A. P. Gould Th. 2.30 |
| Metropolitan Free Hospital, Kingsland Road, N. Sec., Mr. G. Croxton<br>Admission free to the sick poor, without any letter of recommendation. Urgent cases at all times | *In- and Out-Physicians*, Drs. Drysdale, Tu. F. 12; J. G. Dudley, W. S. 12; Fotherby, M. Th. 12; H. H. Tooth, Tu. F. 9; A. Haig, W. S. 9; A. Davies, M. Th. 9<br>*In- and Out-Surgeons*, Messrs. E. J. Chance, W. S. 9; Goodsall, Tu. F. 9; Walsham, M. Th. 9; A. A. Bowlby, F. 9; S. Paget, Th. 9; C. J. Thompson, daily, 9 |
| Middlesex Hospital, Berners Street, W. Sec. and Supt., Mr. A. O. D. Bartholeyns<br>Urgent cases admitted at all times; other cases by ticket. All medical cases must, in the first instance, be seen by the Resident Medical Officer, who attends at the hospital daily, at 11 a.m. | *In-Physicians*, Drs. Cayley, M. Th.; Coupland, Tu. F.; D. Powell, M.Th.; Finlay, Tu. F. Hour, 1.30<br>*In-Surgeons*, Messrs. Hulke, M. Th.; Lawson, M. Th.; Morris, Tu. F. Hour, 1.30<br>*Out-Physicians*, Drs. Fowler, Tu. F. 3.30; Biss, M. Th. 1.30; Pringle, W. S. 1.30<br>*Out-Surgeons*, Messrs. Andrew Clark, M. 1.15 (*Men*); Th. 1.15 (*Women*); Pearce Gould, F. 1.30 (*Men*); Tu. 1.30 (*Women*); J. B. Sutton, W. S. 9 |
| Miller Hospital and Kent Dispensary, Greenwich, S.E. Hon. Sec. Mr. W. Bristow. | *Physicians*, Drs. T. Creed, C. H. Hartt, G. Willes<br>*Surgeons*, Messrs. T. Moore, J. Poland, E. Clarke |
| North-West London Hospital, 18, Kentish Town Road, N.W. Sec., Mr. A. Craske<br>By subscribers' tickets or payment, but accidents and urgent cases free at all times | *In- and Out-Physicians*, Drs. W. C. Hood, J. Shaw, Edwarde H. Campbell. Daily, 1.30<br>*In- and Out-Surgeons*, Messrs. F. Durham, Collier, Jennings, J. Black. Daily, 1.30 |
| Ospedale Italiano, 41, Queen Square, Bloomsbury. Sec., Sig. L. Bonacina<br>Free to all, but Italians have preference | *In- and Out-Physicians*, Drs. M. Castaneda, Tu. F. 10 to 11; B. Sassella, M.Th. 10 to 11; Mr. J.W.B. Steggall, W.S. 4 to 5 |
| Poplar Hospital, 303, East India Dock Road, E. Sec., Lt.-Col. Feneran<br>Free to accidents and emergencies | *In-Surgeons*, Messrs. F. M. Corner, M. Brownfield, and Resident Surgeons<br>*Out-Surgeons*, Dr. J. D. Grant, W. S. Mr. T. E. Bowkett, Tu. F. 12 to 2 |
| Royal Free Hospital, Gray's Inn Road, W.C. Sec., Mr. J. S. Blyth<br>Admission free to the sick poor, without any letter of recommendation | *In- and Out-Physicians*, Drs. Cockle, M. Th.; Sainsbury, Tu. F.; West, W. S. Hour, 1<br>*In- and Out-Surgeons*, Messrs. Rose, M. Th.; W. Gant, Tu. F.; Barrow, W. S. Hour, 1 |
| St. Bartholomew's Hospital, Smithfield. Clerk, Mr. W. Cross<br>Admission free, without letter or ticket. Accidents and other urgent cases admitted at any time; ordinary cases of disease daily, between 9 and 10. Patients are seen for Electrical treatment on M. Tu. Th. and F. 1.30 | *In-Physicians*, Drs. Andrew, Church, Gee, Sir D. Duckworth. Daily, 1.30<br>*In-Surgeons*, Messrs. Savory, T. Smith, Willett, Langton, M. Baker. Daily, 1.30<br>*Out-Physicians*, Drs. Hensley, M. Th. 11; Brunton, Tu. F. 11; Legg, W. S. 11; N. Moore, Tu. W. Th. S. 9<br>*Out-Surgeons*, Messrs. Marsh, Tu. F.; 12; Butlin, M. Th. 12; Walsham, W. S. 12; Cripps and Bruce Clarke, daily, 9<br>*Casualty Physicians*, Drs. Davies, Tu. W. F. S.; O. Browne, M. Tu. Th. F.; Nias, M. W. Th. S. Hour, 9 |
| St. George's Hospital, Hyde Park Corner, S.W. Sec., Mr. C. L. Todd<br>Free to accidents and emergencies. Other in-cases by Governor's or subscriber's letter, but, in necessitous cases, this recommendation is not strictly insisted on. Letters are not required for out-patients. Vaccination on Th. at 11 | *In-Physicians*, Drs. Wadham, M. F.; Dickinson, M. Tu. Th. F.; Whipham, Tu.W. S.; Cavafy, Tu. S. Hour, 1<br>*In-Surgeons*, Messrs. Holmes, M. Th. F. 1, W. 1.30; Rouse, M. Th. F. 1, W. 1.30; Haward, Tu. Th. S. 1, W. 1.30<br>*Out-Physicians*, Drs. Ewart, M. F.; Owen, Tu. S. Hours, 10.30 to 12<br>*Out-Surgeons*, Messrs. Bennett, M. F.; Dent, Tu. S. Hours, 10.30 to 12<br>*Men*, F. S.; *Women*, M. Tu. |

